# Ischaemia–Reperfusion Injury in Organ Transplantation: Role of Coenzyme Q10

**DOI:** 10.3390/jcm14186486

**Published:** 2025-09-15

**Authors:** David Mantle, Neve Cufflin, Tyler T. Purcell, Iain P. Hargreaves

**Affiliations:** 1 Pharma Nord (UK) Ltd., Morpeth NE61 2DB, UK; 2 School of Pharmacy, Liverpool John Moores University, Liverpool L3 5UA, UKi.hargreaves@ucl.ac.uk (I.P.H.); 3 School of Medical Science, University of Manchester, Oxford Road, Manchester M13 9PL, UK; tylerthomaspurcell@gmail.com

**Keywords:** organ transplantation, coenzyme Q10, mitochondrial dysfunction, oxidative stress, inflammation, apoptosis, ferroptosis, stem cells, immunosuppressants

## Abstract

The success of organ transplantation can be compromised by ischaemia–reperfusion injury (IRI), an unavoidable consequence of transplant surgery. IRI is associated with mitochondrial dysfunction, oxidative stress, inflammation, and apoptosis/ferroptosis. There is therefore a rationale for supplementation with coenzyme Q10 (CoQ10) to mediate the adverse effects of IRI, given the role of CoQ10 in promoting normal mitochondrial function, as an antioxidant, and as an anti-inflammatory and anti-apoptotic/ferroptotic agent. In this article we have reviewed the potential role of supplementary CoQ10 in organ transplantation in preclinical animal studies based on the above actions; the role of supplementary CoQ10 in promoting stem cell action in transplantation and its role in alleviating the adverse effects of immunosuppressants used in organ transplantation are also discussed.

## 1. Introduction

The viability of organs for transplant is limited by the time in cold storage, which results in the development of so-called ischaemia–reperfusion injury (IRI). IRI results from the initial interruption of organ blood flow and subsequent restoration of organ blood flow and is a major factor in determining the outcome of organ transplantation. IRI is closely associated with early graft failure, enhancing allograft immunogenicity and promoting acute and chronic rejection. IRI results in mitochondrial dysfunction, with associated oxidative stress, inflammation, and apoptosis/ferroptosis. Mitochondria have a key role in cell metabolism, including the production of ATP, the generation of oxidising free radical species (ROS), the mediation of the immune response, and the regulation of cell death. During ischemia, hypoxia leads to cessation of mitochondrial oxidative phosphorylation (which plays a crucial role in energy production), increased generation of ROS, and initiation of apoptosis/ferroptosis. Reperfusion exacerbates mitochondrial damage, triggering the release of damage-associated molecular patterns (DAMPs) and inflammatory responses. IRI, therefore, comprises a cascade of cellular events, including energy loss, generation of reactive oxygen free radical species, release of cytokines, activation of immune cells, and cell death [1,2]. In this article we have, therefore, reviewed the potential role of coenzyme Q10 (CoQ10) in mediating IRI and promoting the viability of organs for transplantation, given the key role of CoQ10 in normal mitochondrial function, as an antioxidant protecting against ROS-induced cellular damage, and as an anti-inflammatory and anti-apoptotic/ferroptotic agent. The role of supplementary CoQ10 in promoting stem cell action in transplantation, and its role in alleviating the adverse effects of immunosuppressants used in organ transplantation, are also reviewed.

## 2. IRI and Mitochondrial Dysfunction

**Figure 1:**
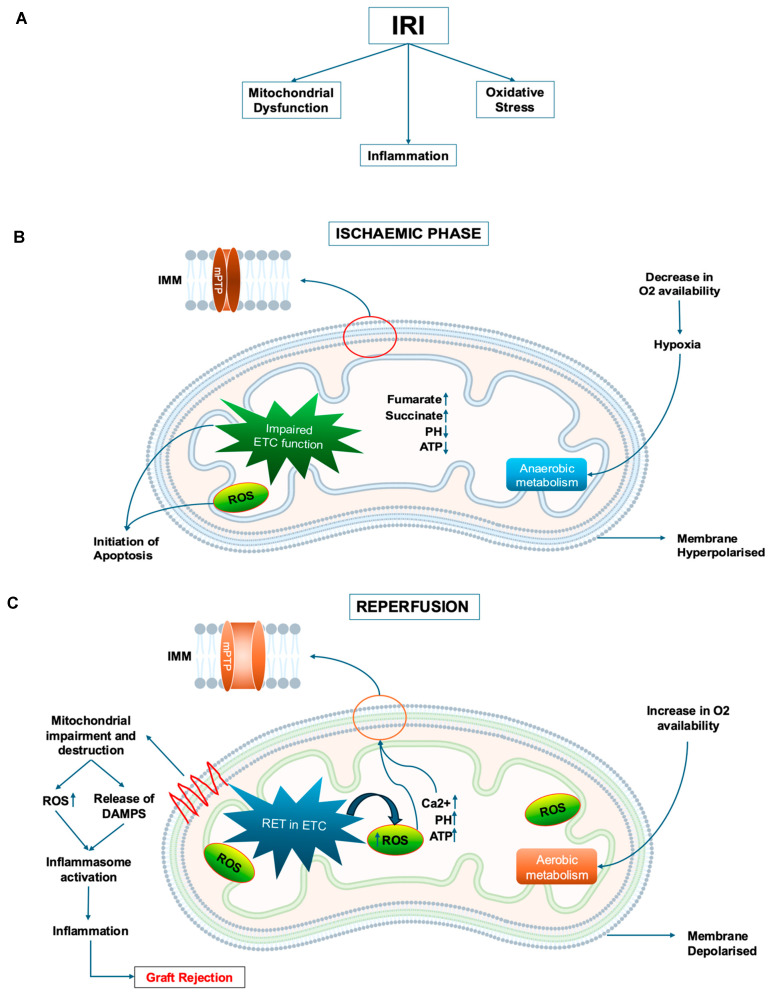
The causes of IRI (**A**) and the involvement of ischaemia (**B**) and reperfusion (**C**) in mitochondrial dysfunction in IRI. IRI: Ischaemia–reperfusion injury. ETC: Electron transport chain. RET: Reverse electron transport. ROS: Reactive oxidative species. mPTP: Mitochondrial permeability transition pore. DAMPS: Damage-associated molecular patterns. ATP: Adenosine triphosphate. IMM: Inner mitochondrial membrane.

As noted in the previous section of this article, IRI results from a self-reinforcing cycle of mitochondrial dysfunction, oxidative stress, inflammation, and cell death. During ischaemia, the transfer of electrons along the mitochondrial electron transport chain (ETC) is disrupted by hypoxia, since during cellular respiration oxygen (because of its high electronegativity) is the terminal electron acceptor in the ETC. This in turn interferes with the transfer of protons across the inner mitochondrial membrane, reducing the proton motive force (i.e., the energy stored as an electrochemical gradient) required for oxidative phosphorylation and adenosine triphosphate (ATP) synthesis. When the blood flow is re-established during reperfusion, re-oxygenation aggravates the damage caused during the ischaemia. The accumulation of fumarate during ischaemic preservation triggers the reverse activity of mitochondrial complex II and the reduction of fumarate to succinate, with succinate accumulating and acting as an electron sink [3]. During reperfusion of the graft, accumulated succinate is rapidly oxidised, triggering reverse electron transport (RET) in complex I, resulting in excess generation of mitochondrial ROS [3]. This in turn results in increased mitochondrial calcium levels and activation of the mitochondrial permeability transition pore (MPTP), resulting in mitochondrial dysfunction, seriously decreasing graft function and survival. In addition, mitochondrial ROS and internal elements from damaged mitochondria (DAMPS), including oxidised mitochondrial DNA (mtDNA), can activate inflammasomes, thereby triggering an innate immune response and accelerating rejection (Figure 1).

A number of studies have provided evidence confirming the importance of mitochondrial dysfunction in IRI. Thus, Pollara et al. [4] demonstrated a correlation between donor plasma mitochondrial DNA levels and early allograft dysfunction in liver transplant recipients, suggesting a role for circulating mtDAMPs in allograft outcomes. Scozzi et al. [5] found high levels of mtDNA in the plasma of patients undergoing lung transplantation were associated with the development of severe graft dysfunction. Lin et al. [6] demonstrated the presence of extracellular mitochondria in the circulation of deceased organ donors and that their presence correlated with early allograft dysfunction; this is a consequence of the mitochondria activating endothelial cells (the initial barrier between a solid organ allograft and its host) to produce inflammatory cytokines. In a series of 61 patients undergoing liver transplantation, Nagakawa et al. [7] reported higher plasma levels of mtDNA DAMPS correlated with a longer duration of post-transplant recovery.

Martins et al. [8] measured changes in mitochondrial function and bioenergetics that occur during ischemia/reperfusion in liver biopsies from a series of 28 patients undergoing liver transplantation. There was a significant reduction in mitochondrial membrane potential, an increase in lag phase, and decreases in mitochondrial respiration and ATP content. Higher postoperative aminotransferase peaks correlated with a worsening of mitochondrial function, and mitochondrial respiration correlated with arterial lactate. Zepeda-Orozco et al. [9] correlated genetic markers of mitochondrial dysfunction with poor graft function in renal biopsies from patients undergoing kidney transplantation. In liver biopsy samples taken after organ cold storage, evaluation of ETC function correlated with clinical outcome following liver transplantation [10]. Following heart transplantation, analysis of endomyocardial biopsies showed impaired myocardial mitochondrial respiration, coupled with myocardial oxidative stress, inflammation, and oedema [11]. Romero et al. [12] reported that cardiac allograft rejection was associated with decreased mitochondrial-related gene expression, following analysis of endomyocardial biopsies from heart transplant patients.

In addition to the above studies using human tissues, evidence of mitochondrial dysfunction in IRI has also been obtained from studies in animal models. For example, Sammut et al. [13] investigated the effect of storage time on hepatic mitochondrial oxygen consumption and activities of ETC complexes I, II, III, IV, and V in mitochondria isolated from rat liver isografts stored for 25 min and 24 h pre- and post-transplantation. The data obtained showed that a loss of membrane integrity, coupled with an inhibition of Complexes I and V and an involvement of Complex II-III in 24-h stored hepatic transplants, accounted for mitochondrial respiratory dysfunction in hepatic transplantation injury. Using isolated rat hearts, Akande et al. [14] demonstrated reduced mitochondrial oxidative phosphorylation and calcium retention following ischaemia, with further exacerbation of mitochondrial dysfunction following reperfusion. In rats, Duboc et al. [15] used NADH laser fluorimetry and mitochondrial oxygraphy to demonstrate impaired myocardial oxidative energy metabolism during cardiac allograft rejection.

As noted in the introduction, CoQ10 has a key role in the normal functioning of mitochondria. CoQ10 has a key role as an electron carrier (from complex I and II to complex III) in the mitochondrial electron transport chain during oxidative phosphorylation. CoQ10 is also a key component in the reactions mediated by other mitochondrial enzymes; for example, it is also involved in the metabolism of pyrimidines, fatty acids, and mitochondrial uncoupling proteins, as well as in the regulation of the mitochondrial permeability transition pore. CoQ10 serves as an important lipid-soluble antioxidant, protecting mitochondrial membranes from free radical-induced oxidative stress [16].

## 3. IRI and Oxidative Stress

Oxidative stress is defined by the imbalance between the production of highly reactive and potentially damaging free radical species (particularly ROS) and the capacity of antioxidants to protect cells against such damage. Within cells free radicals may originate from several sources, but as noted in previous sections of this article, during IRI the principal source of free radicals is from damaged mitochondria. Hypoxia reduces the activity of antioxidant enzymes such as superoxide dismutase, catalase, and glutathione peroxidase; uncoupling of the mitochondrial respiratory chain, together with weakening of the antioxidant system, results in excessive ROS production and oxidative damage to cell components. In addition, enzymes responsible for free radical generation, such as NADPH oxidase and xanthine oxidase, may be activated during IRI, serving as an additional ROS source. The administration of antioxidants in organ donors, organ preservation solutions, and organ recipients has been proposed; however, the results from clinical trials have been equivocal [17].

CoQ10 (particularly in its reduced ubiquinol form) serves as an important lipid-soluble antioxidant protecting cellular membranes, both mitochondrial and extra-mitochondrial (Golgi apparatus, lysosomes, endoplasmic reticulum, and peroxisomes), from free radical-induced oxidative stress (OS). In addition to acting as an antioxidant directly, CoQ10 is also involved in the regeneration of the antioxidants vitamin C and vitamin E, respectively [16].

## 4. IRI and Inflammation

There is a common misconception that inflammation, which involves the release of pro-inflammatory cytokines, is a wholly negative process within the body. However, inflammation is the body’s normal response to infection or injury and is essential for tissue healing, although this process should resolve following the initial immune response. When control of this process is lost, then further tissue damage results; mitochondrial dysfunction and oxidative stress have been identified as factors contributing to the loss of control of the latter process. The dysfunction of mitochondria and the release of danger-associated molecular patterns (DAMPs), ROS, and calcium initiate an immune response involving both innate and adaptive immune systems. The release of mitochondrial DNA (mtDNA) from damaged mitochondria is a particularly potent activator of inflammation [18].

CoQ10 performs several cellular functions of potential relevance to the immune system. Firstly, the immune response has intensive energy requirements, and an adequate supply of CoQ10 is therefore required to enable the various cell types of the immune system to function optimally. Secondly, since phagocytic cells destroy invading pathogens via the production of free radicals, the antioxidant action of CoQ10 may protect phagocytic cells from self-destruction caused by their generation of free radicals. Thirdly, CoQ10 can directly modulate the action of genes involved in inflammation and may have a role in controlling the release of pro-inflammatory cytokines [19].

## 5. IRI and Apoptosis/Ferroptosis

Apoptosis is a tightly regulated process of programmed cell death that can occur as a result of mitochondrial dysfunction following IRI during organ transplantation; pro-apoptotic factors such as cytochrome c are released from damaged mitochondria into the cytoplasm to induce apoptosis mediated by caspase-type proteolytic enzymes [2]. A considerable number of preclinical studies have been reported in which administration of CoQ10 has inhibited apoptosis, for example, after spinal cord injury in rats [20] and in a mouse cell model of diabetes [21].

Another consequence of mitochondrial dysfunction during IRI that can contribute to organ transplant failure is ferroptosis [22]. Ferroptosis is an iron-dependent form of cell death characterised by iron accumulation and extensive lipid peroxidation; it differs morphologically, genetically, and biochemically from other cell death types, including apoptosis. A high level of serum ferritin (as a marker of iron overload) in the donor was found as an independent risk factor for hepatic damage post liver transplantation [23]. Several preclinical studies have demonstrated the action of CoQ10 or its structural analogues in inhibiting ferroptosis; these include models of epilepsy [24], subarachnoid haemorrhage [25], myocardial infarction [26], Parkinson’s disease [27], and acute liver injury [28].

## 6. IRI and Mesenchymal Stem Cells

Mesenchymal stem cells (MSCs), typically derived from bone marrow, have the potential to protect organs from IRI during transplantation via several mechanisms, including antioxidant activity, anti-inflammatory action, anti-apoptotic action, and mitochondrial transfer. A number of studies have demonstrated the potential beneficial effects of MSCs with regard to transplantation of human organs. For example, in lungs from deceased donors undergoing cold storage, intratracheal administration of MSCs significantly decreased the levels of a number of inflammatory markers [29]. Similarly, administration of MSCs to kidneys undergoing normothermic machine reperfusion resulted in reduced inflammation and improved renal function [30].

Several studies have reported the beneficial effects of CoQ10 administration in MSCs. Mauro et al. [31] reported activation of mitochondria in human MSCs following administration of encapsulated CoQ10 using a MITO-Porter mitochondrial delivery system. Using human MSCs, Hernandez-Perez et al. [32] showed a combination of CoQ10 and resveratrol improved the proliferation and differentiation of MSCs and protected against oxidative stress-induced damage. Sun et al. [33] used an emulsified CoQ10 formulation to improve mitochondrial function and cell viability in rat MSCs.

MSC function declines in older individuals, and that MSC dysfunction influences the effects of autologous MSC transplantation in older individuals [34]; in rats, CoQ10 inhibited the ageing of MSCs resulting from intracellular ROS generation induced by D-galactose [35]. Zhang et al. [36] reported that the CoQ10 analogue idebenone promoted MSC proliferation and delayed replicative senescence in rat MSCs. Similarly, Zhong et al. [37] used the CoQ10 analogue MitoQ to reduce oxidative stress-induced senescence in canine MSCs.

## 7. IRI and Mitochondrial Transplantation

A relatively recent strategy to address mitochondrial dysfunction in IRI is mitochondrial transplantation therapy (MTT). MTT uses replacement of defective mitochondria with viable, respiration-competent mitochondria, ideally isolated from non-ischemic tissue obtained from the patient’s own body. The origin of the mitochondria in this context is of primary concern, since, for example, mitochondria in the heart, skeletal muscle, and liver can differ in proteome and function. After injection of mitochondria into the target site, mitochondrial internalisation is largely dependent on cellular macropinocytosis. Studies of MTT in cell culture and animal models have provided promising results, but to date only one MTT-related study has been carried out in human subjects, a phase I clinical trial in paediatric cardiac patients with cardiac IRI [38]. A major limitation of the latter study is that it was a single-centre, non-randomised, retrospective study with historical controls; further randomised controlled trials are therefore required to demonstrate the efficacy and safety of MTT in patients with IRI.

## 8. IRI and Coenzyme Q10

With regard to clinical studies, in a series of patients following heart transplant, Gvodzdjakova et al. [39] reported reduced levels of CoQ10 in both plasma and endomyocardial biopsy samples, correlating with reduced mitochondrial respiratory chain function and the histologically graded extent of transplant rejection. Similarly, Kurcharska et al. [40] found reduced CoQ10 levels in endomyocardial biopsies in patients following heart transplantation. Depleted levels of CoQ10 in heart muscle or plasma were associated with an increased risk of rejection in heart transplant patients [41,42]. Dlugosz et al. [43] reported supplementation with CoQ10 (90 mg/day for 4 weeks) in a series of 11 long-term renal allograft recipients significantly improved the levels of lipid peroxidation/atherogenicity markers.

In addition to mitochondrial dysfunction, another factor that may limit the successful organ transplantation is telomere shortening, when assessment of telomere length in the early post-transplant period allows prediction of long-term function of the transplanted organ [44]. Telomere shortening may be an issue, particularly in the transplantation of older organs [45], although telomere shortening has been suggested to occur as a consequence of IRI/oxidative stress during transplantation surgery [46]. In this regard, it is of note that administration of CoQ10, in combination with selenium, has been reported to reduce telomere shortening in older subjects [47].

Regarding preclinical studies, in a rat model of liver transplantation, pretreatment of the donor rat with CoQ10 (10 mg/kg intravenous) 1 h before surgery was reported to protect against hepatic ischemia induced for 30 min at normothermic body temperature [48]. Using a murine heterotopic cardiac transplantation model, Yuan et al. [49] designed a mitochondrion-targeted nanocarrier loaded with CoQ10 for treatment of cold IRI after cardiac graft. This involved synthesis of hybrid nanoparticles composed of CaCO_3_/CaP/biotinylated-carboxymethylchitosan, followed by incorporation of the mitochondria-targeting tetrapeptide SS31 onto the surface of the hybrid nanoparticles. The SS31 peptide targets the inner mitochondrial membrane by directly interacting with cardiolipin. Donor hearts were perfused with preservation solution containing the above hybrid nanoparticles and stored in vitro at 4 °C for 12 h. The donor hearts were heterotopically transplanted and analysed for graft function, oxidative damage, apoptosis, and inflammatory markers 1 day post-transplantation. The hybrid nanoparticles were shown to localise within mitochondria during cold storage, improving subsequent heart graft function by attenuating mitochondrial oxidative injury and inflammation. In donor dogs, intravenous administration of CoQ10 prior to heart removal maintained tissue ATP levels during organ storage and suppressed the oxidative stress marker malondialdehyde following organ reperfusion [50].

Mitchell et al. [51] reported the addition of the mitochondria-targeted CoQ10 analogue mitoquinone (MitoQ, 100 uM) to the University of Wisconsin organ preservation solution significantly reduced ROS production and mitochondrial dysfunction and improved organ viability in isolated rat kidneys. The same research group also found the addition of MitoQ (100 uM) to University of Wisconsin organ preservation solutions reduced oxidative stress, preserved mitochondrial function, and reduced renal tubular damage during cold preservation of porcine kidneys [52]. Similarly, using a murine heterotopic cardiac transplant model, Dare et al. [53] showed incorporation of MitoQ (50 uM) in Soltran organ preservation solution reduced oxidative damage and dampened the early pro-inflammatory response in the recipient. MitoQ is a conjugate of ubiquinone and the triphenylphosphonium cation, developed to specifically target mitochondria [54]; however, although MitoQ has antioxidant action in common with CoQ10, it differs in other aspects of cell function. The incorporation of the CoQ10 analogue idebenone, which has a much shorter and less lipophilic isoprenyl tail than CoQ10, into University of Wisconsin or histidine–tryptophan–ketoglutarate organ preservation solutions has also been suggested, based on protection against oxidative stress observed in a rat liver microsomal model [55] and reduced heat shock protein expression in isolated perfused pig liver [56]. However, in an animal study using liver submitochondrial particles, idebenone treatment was reported to inhibit the activity of ETC complex I [57]. Therefore, in addition to its therapeutic capacity, idebenone may also have the potential to induce ETC dysfunction.

There is considerable evidence, from clinical and particularly preclinical studies, for the efficacy of supplemental CoQ10 in mediating IRI in situations other than organ transplantation. For example, in patients undergoing elective coronary artery bypass surgery, pretreatment with CoQ10 reduced oxidative stress following cardiopulmonary bypass and aortic cross-clamp removal; the incidence of ventricular arrhythmias during the recovery period was also reduced compared to non-supplemented controls [58]. In patients with myocardial infarction undergoing primary percutaneous coronary intervention, supplementation with CoQ10 reduced plasma levels of oxidative stress biomarkers resulting from IRI [59]. The outcomes of preclinical studies supplementing CoQ10 in a variety of animal models of IRI are summarised in Table 1. In most of these studies, animals were typically pre-dosed (oral or intravenous unless otherwise indicated) with CoQ10 prior to the operative procedure; studies used the ubiquinone form of CoQ10, unless indicated otherwise in Table 1.

## 9. CoQ10 and Immunosuppression

Tacrolimus is a widely used immunosuppressive agent for the prevention of allograft rejection after renal transplantation. However, major adverse effects of long-term administration of tacrolimus are progressive renal failure and new-onset diabetes mellitus. Using a rat model of tacrolimus-induced nephropathy, Yu et al. [81] found administration of CoQ10 (20 mg/kg/day for 4 weeks by oral gavage) reduced oxidative stress and improved both mitochondrial function and renal function. Similarly, in a rat model of tacrolimus-induced diabetes, Luo et al. [82] reported administration of CoQ10 (20 mg/kg/day for 4 weeks by oral gavage) reduced oxidative stress, improved mitochondrial ultrastructural parameters and respiratory function, and improved pancreatic beta cell function. Yang and colleagues used a water-soluble formulation of CoQ10 (based on a CoQ10/eicosapentaenoic acid/glycyrrhizin nanoemulsion formulation) to reduce oxidative stress, improve mitochondrial ultrastructure, and improve tissue function in rat models of tacrolimus-induced nephropathy [83] and tacrolimus-induced diabetes mellitus [84], respectively. Oral administration of CoQ10 (20 mg/kg/day for 2 weeks) has also been shown by the same research group to reduce oxidative injury and restore mitochondrial ultrastructure in pancreatic islets in diabetes mellitus induced in rats by the related immunosuppressive agent sirolimus [85]. In the above studies by Yang and colleagues, oxidative stress was quantified via measurement of 8-hydroxydeoxyguanosine and 4-hydroxyhexenal levels as biomarkers of oxidative damage to DNA and lipids, respectively.

Cyclosporine and cyclophosphamide are immunosuppressants used in organ transplantation, with renal or cardiac injury as an adverse effect. Supplementation with CoQ10 (5 mg/kg/day for 28 days) partially prevented cyclosporine-induced cardiotoxicity in rats [86]. Sato et al. [87] reported administration of CoQ10 (in ubiquinol reduced form, 600 mg/kg/day for 4 weeks) reduced oxidative stress (quantified via 8-hydroxydeoxyguanosine level) and renal function (assessed via urinary albumin and serum creatinine levels) in a rat model of cyclosporine nephrotoxicity. In rats, administration of CoQ10 (in ubiquinol form, 600 mg/kg/day for 4 weeks) reduced cyclosporine nephrotoxicity, assessed via serum creatinine and urinary albumin levels [88]. Administration of CoQ10 reduced oxidative stress (assessed via blood malondialdehyde level) and tissue damage (assessed histologically) in a rat model of cyclophosphamide-induced renal injury [89]. It is of note that oral administration of CoQ10 (10 mg/kg for 10 days) reduced cyclophosphamide-induced cognitive and motor dysfunction in rats by reducing oxidative injury, repressing intrinsic apoptosis, boosting neurogenesis, and upregulating the Wnt/β-catenin pathway [90]. CoQ10 administration has been shown to ameliorate cognitive impairment in animal models induced by other types of immunosuppressant agents, of relevance to cancer therapy [91].

## 10. Discussion and Conclusions

Ischaemic reperfusion injury is an unavoidable consequence of organ transplantation. In the present article, we have reviewed evidence for the involvement of mitochondrial dysfunction, oxidative stress, inflammation, and apoptosis/ferroptosis in IRI during transplantation, with consequences for transplanted organ failure. These data provide a rationale for the potential role of coenzyme Q10 in mediating IRI and promoting the viability of organs for transplantation, given the key role of CoQ10 in normal mitochondrial function, as an antioxidant protecting against ROS-induced cellular damage, and as an anti-inflammatory and anti-apoptotic/ferroptotic agent. One of the principal ways in which CoQ10 mediates oxidative stress, inflammation, and apoptosis/ferroptosis is via its interaction with intracellular signalling pathways, most notably the Nrf2/NQO1/HO1, NF-κB, and P13K/AKT/mTOR pathways. Nuclear factor erythroid 2-related factor 2 (Nrf2) is a transcription factor that acts as a master regulator of genes controlling the production of antioxidant enzymes, such as NAD(P)H quinone dehydrogenase 1 (NQO1) and heme oxygenase (HO). Supplementary CoQ10 increases the levels of Nrf2, subsequently increasing antioxidant expression [92]. The nuclear factor kappa-light-chain-enhancer of activated B cells (NF-κB) pathway regulates the immune response and inflammation, with NF-κB comprising a family of transcription factors regulating the expression of pro-inflammatory cytokines. An example of the suppression of the NF-κB pathway by CoQ10 is the reduction in inflammation induced by beta-amyloid in nerve cells [93]. The P13K/AKT/mTOR pathway is involved in the promotion of cell survival by inhibiting apoptosis, with P13K, AKT, and mTOR referring to different types of kinase enzymes involved in the pathway [94].

Depletion of tissue CoQ10 levels has been associated with a variety of disorders, and the question arises as to how CoQ10 depletion may impact organ transplantation. To assess CoQ10 status, a plasma determination may be undertaken. However, the CoQ10 status of plasma has been reported to be influenced by both dietary supply and hepatic biosynthesis [95] and therefore may not reflect tissue levels. Assessment of blood mononuclear cells (MNCs) has been suggested as an alternative surrogate to evaluate endogenous CoQ10 status [95]. MNCs can be easily isolated from EDTA/Li-Heparin blood, and the CoQ10 status of these cells has been reported to correlate with that of skeletal muscle [95]. Studies in human subjects have demonstrated that depleted levels of CoQ10 in plasma or heart biopsy tissue are associated with an increased risk of rejection in heart transplant patients; however, to date no randomised controlled clinical trials supplementing CoQ10 in patients undergoing organ transplantation have been reported, and this remains an area for future research. In this regard, the safety of CoQ10 administration is well established; more than 200 randomised controlled clinical trials are currently listed on Medline in which supplementary CoQ10 has been administered in a variety of disorders in various dosages (up to 3000 mg/day) and for various time periods (up to 5 years); in none of these studies were any serious adverse effects attributable to CoQ10 reported. Preclinical studies have demonstrated that pretreatment of donor animals with CoQ10 helps to protect against IRI during subsequent organ storage. With regard to a related issue, a number of studies in animal models have reported a beneficial effect of supplemental CoQ10 in reducing toxic effects of immunosuppressant drugs such as tacrolimus, cyclosporine, and cyclophosphamide. Similarly, there is evidence for their action of CoQ10 in promoting the metabolism of stem cells, which in turn may be utilised to benefit the transplantation process.

Although the present review has focused on IRI and organ transplantation, factors other than IRI may be involved in transplantation outcome. Such factors include graft-versus-host disease (GVHD), which occurs when donor immune cells attack tissues of the recipient, and may involve mitochondrial dysfunction, oxidative stress, and inflammation in the latter [96]. It is of note that supplementation with CoQ10 has been reported to reduce the severity of GVHD in a mouse model of this disorder [97], so this is yet another area relating to transplantation and CoQ10 supplementation requiring further research.

In summary, as listed in Table 1, there is good evidence from preclinical models for the efficacy of supplemental CoQ10 in protecting against IRI generally and, to a lesser extent, against IRI specifically in organ transplantation. While there is therefore a rational basis for considering CoQ10 supplementation to protect against IRI during human organ transplantation, an appropriate randomised controlled trial is now required before supplementary CoQ10 could be recommended for surgical practise.

## Figures and Tables

**Table 1:** Summary of preclinical studies supplementing CoQ10 in animal models of IRI.

Outcomes Following CoQ10 Supplementation	Model System	Study
Myocardial stunning time reduced	Cardiac ischaemia and reperfusion in pigs	Atar et al. [60]
Reduced oxidative stress and improved cardiac function	Cardiac ischaemia and reperfusion in rats	Niibori et al. [61]
Reduced oxidative stress and improved cardiac function	Cardiac ischaemia and reperfusion in pigs	Maulik et al. [62]
Reduction in irreversibly damaged myocardium	Cardiac ischaemia and reperfusion in rabbits	Verma et al. [63]
Reduced oxidative stress and improved bladder function	Effect of ischaemia–reperfusion on bladder in rabbits	Juan et al. [64]
Reduced oxidative stress and apoptosis	Testicular ischaemia–reperfusion in rats	Erol et al. [65]
Reduced oxidative stress; improved histologic evaluation scores	Ischaemia–reperfusion in rat ovary; intraperitoneal injection of CoQ10	Ozler et al. [66]
Reduced oxidative stress, reduced inflammation, and improved renal morphology	Effect of ischaemia–reperfusion on kidneys in rats; ubiquinol form of CoQ10 supplemented	Peerapanyasut et al. [67]
Reduced oxidative stress, greater preservation of motor neurons, and improved neurological function	Spinal cord ischaemia–reperfusion in rats	Hwang et al. [68]
Improved flap survival rate	Effect of ischaemia–reperfusion on epigastric flap in rats	Ozalp et al. [69]
Reduced cerebral infarct volume and improved neurological behaviour	Cerebral ischaemia–reperfusion in rats	Belousova et al. [70]
Reduced oxidative stress, reduced apoptosis, and improved cardiac function	Cardiac ischaemia–reperfusion in rats; intraperitoneal injection of CoQ10	Liang et al. [71]
Reduced interstitial oedema, degeneration of muscle fibres, and infiltration of mast cells via inhibition of the NF-κB pathway	Ischaemia–reperfusion of skeletal muscle in rats; intraperitoneal injection of CoQ10	Boroujeni et al. [72]
Reduced infarct volume and improved neurological function	Cerebral ischaemia–reperfusion in hyperglycaemic rats	Lu et al. [73]
Reduced apoptosis and improved retinal ganglion cell survival	Ischaemia–reperfusion of mouse retina; ubiquinol form of CoQ10 supplemented	Ju et al. [74]
Reduced oxidative stress, inflammation, and apoptosis; improved renal function	Renal ischaemia–reperfusion in rats	Akbulut et al. [75]
Reduced oxidative stress, inflammation, apoptosis, and mtDNA damage; improved renal function	Renal ischaemia–reperfusion in mice	Liu et al. [76]
Reduced oxidative stress, inflammation, and apoptosis	Testicular ischaemia–reperfusion in rats	Ayengin et al. [77]
Reduced brain oedema and improved cognitive function	Cerebral ischaemia–reperfusion in rats	Fatemi et al. [78]
Reduced oxidative stress and inflammation; reduced cerebral tissue damage	Cerebral ischaemia–reperfusion in rats	Fakharaldeen et al. [79]
Reduced oxidative stress and ferroptosis	Hepatic ischaemia–reperfusion in mice	Guan et al. [80]

## Data Availability

Not applicable.
